# Field sampling marine plankton for biodiscovery

**DOI:** 10.1038/s41598-017-15980-8

**Published:** 2017-11-20

**Authors:** Richard Andre Ingebrigtsen, Espen Hansen, Jeanette Hammer Andersen, Hans Christian Eilertsen

**Affiliations:** 1 Norwegian College of Fishery Science, UiT - The Arctic University of Norway, 9019 Tromsø, Norway; 2 Marbio, UiT - The Arctic University of Norway, 9019 Tromsø, Norway

## Abstract

Microalgae and plankton can be a rich source of bioactivity. However, induction of secondary metabolite production in lab conditions can be difficult. One simple way of bypassing this issue is to collect biomass in the field and screen for bioactivity. Therefore, bulk net samples from three areas along the coast of northern Norway and Spitsbergen were collected, extracted and fractionated. Biomass samples from a strain of a mass-cultivated diatom *Porosira glacialis* were used as a reference for comparison to field samples. Screening for bioactivity was performed with 13 assays within four therapeutic areas: antibacterial, anticancer, antidiabetes and antioxidation. We analysed the metabolic profiles of the samples using high resolution - mass spectroscopy (HR-MS). Principal component analysis showed a marked difference in metabolite profiles between the field samples and the photobioreactor culture; furthermore, the number of active fractions and extent of bioactivity was different in the field compared to the photobioreactor samples. We found varying levels of bioactivity in all samples, indicating that complex marine field samples could be used to investigate bioactivities from otherwise inaccessible sources. Furthermore, we hypothesize that metabolic pathways that would otherwise been silent under controlled growth in monocultures, might have been activated in the field samples.

## Introduction

When investigating marine organisms for bioactivity, it is common to invest considerable time and resources into sorting samples to species level before preparing extracts for further analysis. Commonly, this approach has been applied to benthic organisms^1^, and microorganisms such as bacteria, fungi and microalgae that can be isolated and cultivated^2,3^. Cultivation, however, fails to emulate a natural situation where microorganisms are affected by variable abiotic and biotic stressors that may induce production of secondary metabolites^4^. Genome sequencing also indicates that microalgae, such as diatoms, are genetically diverse and could have the potential to produce a large number of secondary metabolites^5,6^. The majority of all microbial biodiversity is so far regarded as uncultivable; consequently, large parts of the chemical profile of pelagic communities may be “lost”^7^. Since bioactive secondary metabolites may remain unexpressed or under - expressed in monocultures, researchers have sought alternative approaches to access these metabolites. New technologies and techniques targeting naturally expressed bioactivity, such as synthetic membrane technologies and *in situ* cultivation (in the original place) have the potential to push the boundaries of microbial cultivability^8,9^. *In situ* cultivation can present challenges, as well as new opportunities when searching for novel compounds in the sea. In “bulk-sampling” approaches, aimed at targeting complete organism communities, it may prove difficult to identify the specific producer of a desirable secondary metabolite or to obtain large amounts of a sample. In addition, optimal growth conditions can obviously be challenging to replicate if the bioactivity results from a rare community ephemeral situation. However, if successful isolation of a microbe and subsequent characterisation and structural determination of a compound is achieved, it may give valuable information on the bioactivity of pelagic microorganism communities. Alternatively, it is sometimes possible to synthesize compounds responsible for such bioactivity, allowing further investigations that alleviate the need to identify and harvest more of the producing organism^10^.

The Barents Sea hosts one of the most productive marine ecosystems and some of the most productive fishing grounds in the world^11^. At the base of this food web are a plethora of phytoplankton species, fuelling the system with vast amounts of energy in the form of lipids, proteins and sugars. A large part of this energy sequestering and transfer happens during the intense and short - lived spring bloom^12^. These blooms are dominated by few taxonomic clades, e.g. members of the species rich diatom group (Bacillariophyceae) and the haptophycean *Phaeocystis pouchetii* ^13^. However, the diatom group is a diverse assemblage of species, and although abundant, they still form complex plankton communities together with a large number of other planktonic species. In these complex communities, phytoplankton thrive and are able to successfully reproduce in numbers so large that at peak bloom their presence is visible even from space^14^. Phytoplankton naturally has a large number of adaptations that make it possible for them to flourish in the pelagic realm when conditions are favourable. Many behavioural processes are interfaced by chemical cues ranging from feeding and predator deterrence to communication, mating and synchronization. Today there is a growing understanding of the important role that chemical ecology and allelopathy, i.e. chemical influence, plays in the interactions within and between species, and ultimately in the structuring of whole ecosystems^15,16^. The allelopathic properties of phytoplankton-sourced chemicals suggests that phytoplankton are not merely small self-replicating, energy sequestering packages of food for their grazers to consume, but have a great role in structuring ecosystems^17–19^. Some of these allelopathic adaptations are the potential to produce toxins^20^. But, pheromones and signal molecules, allowing synchronization of certain “behaviours”, may also explain some of their remarkable ecological successes^21^. This chemical diversity can also be interesting in terms of natural product discovery.

Natural products, i.e. compounds found in nature, are very important in drug discovery^22^. Most of the drugs from natural sources stem from terrestrial plants or microbes^23^. During the last decades there has been a growing interest, and some successes in searching the marine realm for new bioactive compounds with a wide range of medical or other potentials^24–26^. These include for example: antidiabetes, anticancer, antibacterial, anti-inflammatory, antifungal, antiviral and so on^27–30^. Despite the potential for biodiscovery within e.g. diatoms, and corresponding reports on their respective bioactivity^31–37^, few reports of novel compounds from diatoms exist. In the MarinLit database^38^, of the >27000 compounds of marine origin were described as of 2015; only 22 of these compounds were originally detected in diatoms^39–47^. The relatively few records of diatom compounds could possibly be ascribed to under-sampling, or alternatively, to a relatively small potential to synthesize complex bioactive compounds.

The aim of this study was therefore to investigate bulk plankton samples from three environmentally different areas for therapeutically - relevant bioactivity. Specifically, we aimed to collect mixed plankton communities with plankton nets, subsequently prepare extracts from these samples and ultimately directly test for bioactivity. This work is important both for biodiscovery potential and for overcoming challenges associated with large - volume microalgae monocultures.

## Methods

### Study area and sample collection

**Figure 1:**
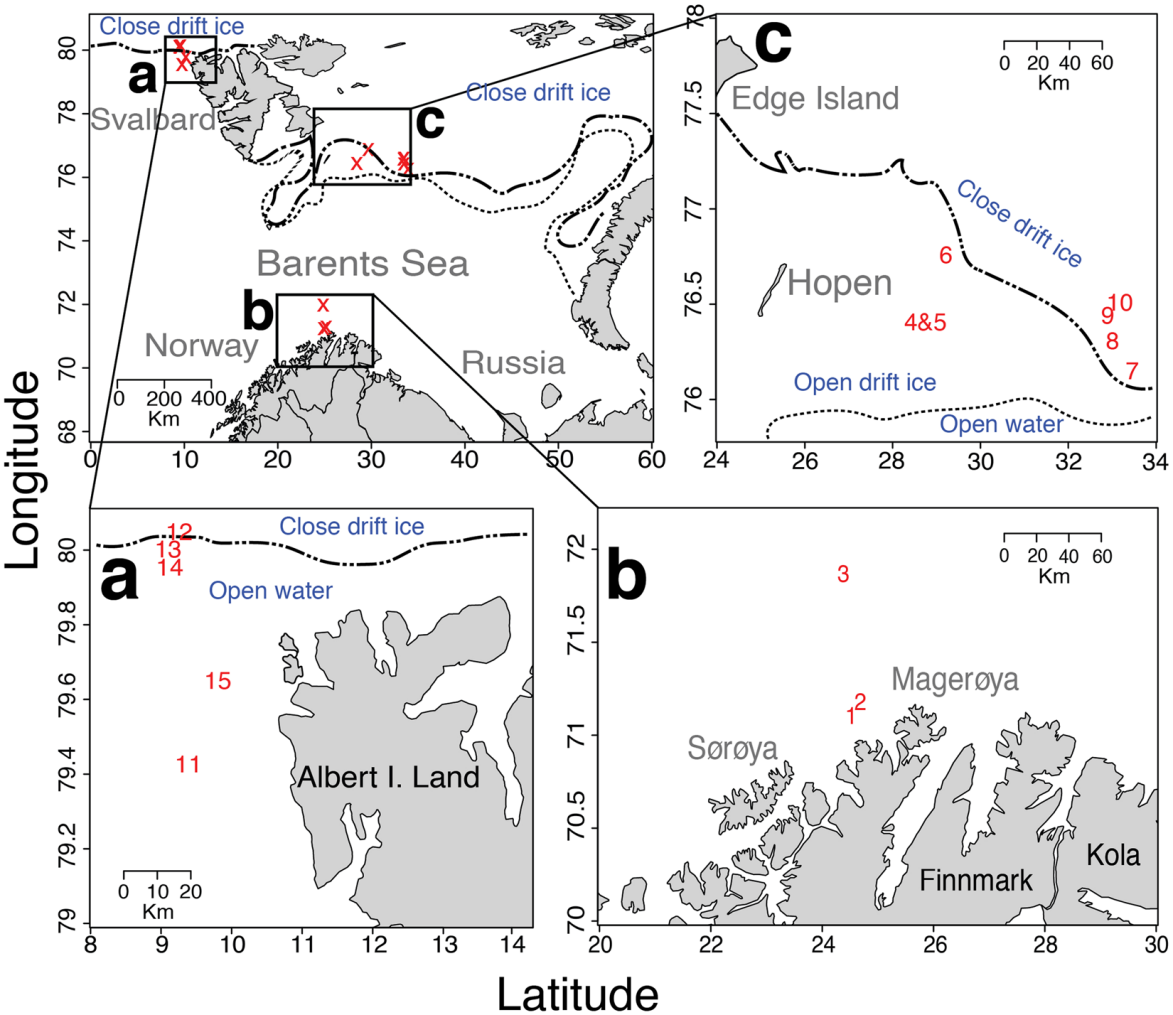
Map showing the three sites sampled (**a**,**b** and **c**) where the WP-2 net samples were collected. The numbers in the maps indicates station numbers and positions. The ice edge was adapted from a May 7th ice map retrieved from The Norwegian Metrological Institutes Sea Ice Service Forecasting Division For North Norway (met.no). Please note the different spatial scale in the panels. The map was created using R version 3.2.2^52^ with the package “Maps”^55^ (https://CRAN.R-project.org/package=maps).

During a research cruise aboard the R/V Helmer Hanssen (28^th^ April to 15^th^ May 2014), a total of 15 plankton biomass samples were collected from three separate areas, i.e. the coast of Finnmark, east of Hopen Island and north west of Svalbard. We collected one sample from each station and sampled: three stations from Finnmark, seven stations from Hopen and five stations from north west of Svalbard (see Fig. 1). We used a standard vertical plankton net, WP - 2, with an opening area 0.25 m^2^, mesh size 180 *μ*m and a 5 L cod-end. To sample the water columns, the net was hauled from 50 to 0 meters depth. Immediately after collection, the resulting 5L samples were decanted slowly for (ca. 10 minutes) into a 20 μm plankton net and frozen at −23 °C. Please note that station 4 and 5 was one net haul, split into a phytoplankton (station 4) and zooplankton (station 5) component by sieving out (400 *μ*m mesh) zooplankton prior to the decanting step. We collected physical oceanographical data (temperature, conductivity and density) and *in vivo* chlorophyll *a* using a Seabird 911+ CTD (Seabird Inc.). Phytoplankton abundance data and species composition were recorded at all stations. Water samples were collected with Niskin 5-L water bottles at 0, 5, 10, 20, 50, 100 meters depth and 10 m above the bottom (if deeper than 100 meters). Live phytoplankton samples from all sampled depths were enumerated and identified to taxonomic species, genus or class on-board using inverted Zeiss Labovert microscopes at 400x magnification and Nunclon 2 mL 4-well chambers (one chamber for each depth). For taxonomy we followed Hasle and Syvertsen^48^. As a quality control check, we performed qualitative microscopy of WP-2 samples to compare cell abundance and composition to samples observed in the Niskin samples. *In vitro* chlorophyll *a* was determined using a calibrated TD 700 fluorometer (Turner Designs) according to the method of Holm-Hansen and Riemann^49^, though using 96% EtOH as extractant. Phaeophytin was determined by measuring the sample after adding one droplet of 10% HCl.

### Mass cultivation of *Porosira glacialis*

Biomass of the northern marine diatom *Porosira glacialis* was used as a standard for comparing biomass of multi-species field samples. Three samples from a batch cultivation of a non-axenic *P. glacialis* monoculture in exponential growth phase were collected (sample a, b and c, ca. 24 hours between each harvest). The *P. glacialis* strain was cultivated outdoors in a 6000 L stainless steel tank with extra LED light illumination giving ca. Ø 45 μmol photons m^−2^ s^−1^. The light period was set to 14:10 (light: dark cycle). Seawater for cultivation was filtered with a ceramic membrane filter with 0.2 μm pores (Nordisk Vannteknikk). Nutrients were added: 0.25 mL/L^−1^ Substral household plant fertilizer (Scotts Nordic) and 12.3 μmol Si(OH)_4_ L^−1^ silicate solution (Sigma Aldrich). Harvesting was done using a 20 μm mesh plankton net. The wet biomass was centrifuged at 3000 RPM for 5 minutes to remove excess water and the pellet was subsequently frozen at −23 °C.

### Sample preparation

The concentrated WP-2 samples were freeze-dried and extracted overnight at 4 °C in 80% aqueous methanol (Merck methanol diluted with ultra pure water Milli-Q, Millipore). Each extract was filtered through Whatman no. 3 filters (ø 125 mm), and material collected onto the filter was re-extracted overnight (once). The filtrates were stored at 4 °C. The filtrates were combined and reduced to dryness using rotavapors under reduced pressure at 40 °C (Laborota 4002, Heidolph), and the dried extracts were stored at −23 °C.

### Fractionation and sample preparation

Subsamples of the extracts (approximately 1.5 grams) were Flash – fractionated on a Biotage™ SP4 Flash chromatography system (Charlottesville, USA) into eight fractions using gradients of MilliQ – water, methanol and acetone (see Supplementary Table S1). Hereafter, the fractionated extracts will be referred to as fractions. Flash - columns were packed with 6.5 g Diaion^®^ HP20SS resin (Supelco; USA). After fractionation, the eight fractions were reduced to dryness under reduced pressure with a rotavapor at 40 °C (Laborota 4002; Heidolph) and weighed. The dry fractions were dissolved in 25 µL 100% DMSO (Merck) per mg sample (i.e. 40 mg/mL), and aliquots of 25 µL sample solution (i.e. 1 mg sample) were transferred to deep well plates for screening, while the surplus was stored at −20 °C for later use. Prior to screening, the samples were diluted with 975-µL dH_2_O, to a concentration of 1 mg/mL. All assays were tested for DMSO tolerability, and total DMSO concentrations used in screenings were well below the tolerability levels for the assay (DMSO tolerability levels: bacteria <1%, cell lines < 0.5%, chemical assay <5%).

### HR-MS analysis

The dried extracts were resuspended in 100 µL 80% methanol and analysed by an Acquity UPLC and a LCT Premier time-of-flight mass spectrometer (Waters, Milford, MA, USA) using a Waters Acquity BEH C18 column (1.7 µm, 2.1 × 50 mm) operated at 40 °C. The samples were ionized using positive electrospray (ESI+) and the mass range was set to *m/z* 100–1500 and the acquisition time was 0.15 s. The capillary and sample cone voltages were set to 2.9 kV and 50 V (respectively), and the desolvation and ion source temperatures were set to 300 and 120 °C (respectively). Nitrogen was used as desolvation gas at a flow rate of 650 L h^−1^. The MS was tuned to a resolution of 11 000 (FWHM), calibrated with sodium formate, and leucine-enkephaline was used as Lock Mass. Water (A) and acetonitrile (B), both containing 0.1% formic acid, were used as mobile phases at a flow rate of 450 µL min^−1^. 5 µl of the extracts were injected on the column and eluted with the following gradient: 0–0.5 min hold at 30% B, 0.5–4.5 min linear gradient 30–95% B, 4.5–6 min hold at 95% B. Four injections of each extract were analysed.

### MS data analysis

The HR-MS data were analysed using MassLynx 4.1 and the MarkerLynx application manager (Waters). Markers between 150 and 1500 Da were collected with an intensity threshold of 500 counts and retention time and mass windows of 0.2 min and 0.1 Da, respectively. The noise level was set to 10.00 and the raw data were de-isotoped.

### Antibacterial assays

To assess the antibacterial activity in the fractions, we tested against a panel of five different bacterial strains. The bacterial strains were seeded from blood agar into 8 mL of cultivation medium. *Enterococcus faecalis* (ATCC – 29212) and *Streptococcus* group B (ATCC – 12386) were cultivated in brain heart infusion media (BHI; Becton-Dickinson), while *Escherichia coli* (ATCC – 25922), *Staphylococcus aureus* (ATCC – 25923), *Pseudomonas aeruginosa* (ATCC – 27853) was cultivated in Mueller – Hinton broth (MH; Becton - Dickinson). After 24 hours the bacterial suspensions were transferred onto fresh medium until they reached log-phase growth. When in log-phase, cultures were diluted 1:1000 with the appropriate enrichment medium. 50 µL of the diluted bacteria suspensions were then added to each well in clear 96 – well plates (one bacteria strain per plate). Fractions were screened at a concentration of 250 µg/mL. Incubations lasted 24 hours at 37 °C. If the fractions visibly colored the wells, then absorbance was measured prior to addition of bacteria - suspension and post compensation was performed. Negative control was 50 µL enrichment media with 50 µL sterile water. The positive control consisted of 50 µL sterile water with 50 µL of bacterial suspension. Gentamicin (10 mg/mL, Aventis Pharma) was added as a control. Visible growth inhibition was evaluated and in addition a VICTOR^3^ 1420 Multilabel Counter (Perkin Elmer) was used to read absorbance at 600 nm. Activity threshold (referred to as “active” in results) was set to be OD_600_ absorbance values below 0.05 as read in a 1420 Multilabel Counter VICTOR^3^, while “weak active” was OD_600_ values between 0.05–0.09.

### Anti-biofilm assay

In order to investigate activity against biofilm formation, we tested the Flash - fractionated extracts against *Staphylococcus epidermidis* (ATCC – 35984). Bacteria were seeded from blood agar into Tryptic Soy Broth enrichment media (TSB; Merck) and incubated over night before being diluted 1:100 with TSB with 1% glucose. Fractions were tested in triplicates at a concentration of 100 µg/mL. 50 µL fraction and 50 µL bacterial suspension were added to each well in 96-well microtiter plates. *Staphylococcus haemolyticus* (Clinical isolate 8–7 A) was used as a negative control while the blank consisted of enrichment media and sterile water (1:1). The microtiter plates were incubated overnight at 37 °C. The bacterial suspensions were then washed off before fixation of biofilm at 55 °C for 1 hour and staining with 0.1% crystal violet solution. After drying, the plates were visually evaluated for anti - biofilm formation activity. If clear inhibition of biofilm formation was seen in two or three of the triplicates, the fraction was listed as “active”. 70 µL EtOH was added to the wells to dissolve crystal violet before reading absorbance in a VICTOR^3^ 1420 Multilabel Counter (Perkin Elmer) at 600 nm.

### Viability assays

The Flash - fractions were tested against the adherent human melanoma cell line A2058 (ATCC: CRL-11147TM) and adherent normal lung fibroblast cell line MRC5 (ATCC: CCL-171). Cells (ca. 2000 cells/well) were seeded onto transparent 96-well microtiter plates with Roswell Park Memorial Institute 1640 medium with 10% foetal bovine serum and 10 mg/mL gentamicin (RPMI, Biochrom, Germany). The microtiter plates were incubated in the dark overnight at 37 °C with 5% CO_2_ and humidified air before exposure to Flash - fractions, and subsequently incubated 72 hours. Fractions were added in triplicates. Cells treated only with medium were used as a negative control, while cells treated with Triton X-100 (Sigma-Aldrich) at a concentration of 0.5% (resulting in 0% survival) were used as positive controls. Cell viability was determined using the colorimetric MTS assay as follows: At the end of incubation, 10 µL CellTiter 96 Aqueous One Solution Reagent (Promega, USA) was added to each well before an additional hour of incubation. Absorbance was calculated using a DTX 880 Multimode Detector (Beckman Coulter) at 485 nm and the activity threshold was set to be below 50% cell survival, while weak active was just above this threshold (typically 1–10%) compared to the positive and negative control.

### Anti- cancer kinase assay

The ability of the fractions to inhibit the phosphorylation activity of the kinases PKA and ABL was investigated using the Kinase Reaction Rate kit (BioThema AB, Handen, Sweden) according to manufacturer’s protocol. The kit gives a real – time reaction value based on bioluminescence generated in a luciferin/luciferase reaction. Assay was run in white, 384-well flat-bottom OptiPlates (PerkinElmer, MA, USA) and the luminescence was measured after 5 min and 1 hour with an Envision Luminescence reader (PerkinElmer). Active mix with staurosporine at 1 µM concentration was used as positive control, while negative control was active mix with 2.5% DMSO (Merck)/MilliQ -water.

### Diabetes assay

To test for anti-diabetic effect, we applied the enzymatic human recombinant protein tyrosine phosphatase 1B (PTP1B, Calbiochem) assay using the fluorescent substrate 6,8 - difluoro-4-methylumbelliferyl phosphate (DiFMUP, VWR, Leuven, Belgium). Activity is proportional to fluorescence. The assay buffer consisted of 25 mM Hepes, 50 mM NaCl, 2 mM Dithriothethreiol, 2.5 mM EDTA and 0.01 mg/mL Bovine Serum Albumine (BSA). Assay buffer was used as negative control. The positive control consisted of a 160 µM solution of PTP inhibitor IV (Calbiochem) in assay buffer. Aliquots with 25 µL fractions and 50 µL (1.56 ng per well) PTP1B were added to wells in black, flat - bottom 96 - well microtiter plates and incubated in the dark at 37 °C for 30 min. After, 25 µL 10 µM DiFMUP was added and the plates incubated in the dark for 10 min. Fluorescence was measured using a DTX 880 Multimode Detector (Beckman Coulter, USA) with excitation wavelength at (λ) 360 nm and emission at 465 nm. Inhibition was calculated by comparing measurements with controls. “Active” threshold was set to be below 30% activity.

The PTP1B assay is very sensitive, and specificity against PTP1B was checked using the T – Cell -PTP counter screen assay. TC - PTP is essential for life and very similar to PTP1B, so if inhibited, the hit will not be followed up. The assay is similar to the PTP1B assay, except using TC-PTP (R&D Systems, 1930-PT), instead of PTP1B.

### Antioxidant assays

#### Cellular antioxidant assay (CAA)

Some antioxidants can traverse cell membranes; therefore we applied a fluorescent probe assay to investigate the intracellular antioxidant activity caused by the fractionated extracts. Any intracellular antioxidant activity caused by components in the content of the fractions against free radicals was determined using HepG2 liver epithelium cells exposed to 200 mM AAPH (Cayman), to create radicals, and compared to an antioxidant-control of 50 µg/mL of the antioxidant Luteolin (Cayman). Negative control was Hanks saline solution (Biochrom) without AAPH, while positive control was Hanks saline solution with 200 mM AAPH (Cayman), adapted from Wolfe and Liu^50^. Around 80.000 HepG2 cells per well were seeded in black 96 –well microtiter plates (Costar, Cornings). The plates were incubated for 24 hours at 37 °C with humidified air and 5% CO_2_. To1 L E-MEM (Biochrom) growth medium it was added100 mL 10% fetal bovine serum, 1 mL Gentamicin solution (10 mg/mL, Amresco), 10 mL non-essential amino acids (Biochrom), 10 mL sodium pyruvate (Biochrom) and 10 mL L-glutamine (Biochrom). The treatment medium was without FBS, and was added 1.56 µL/mL of 20 mM DCFH-DA solution (2,7 dichlorofluorescine dacetate, Fluka). Fluorescence was read at (λ) 485/520 nm (excitation/emission) with a Perkin Elmer 1420 Victor plate reader using Workout 2.0 software from Dazaq Solutions Ltd. The activity threshold was set to: active = <50% activity, weak active = 50–60% while inactive >60%.

#### Cellular lipid peroxidation assay (CLPAA)

The cellular lipid peroxidation activity assay (CLPAA) was used to measure lipid oxidation in living cells using C11-BODIPY (Invitrogen). The method is adapted from Pap, *et al*.^51^. HepG2 cells were seeded in black 96 well microtiter plates (Costar). Medium used was E-MEM (Biochrom) with the same additives as when used with the CAA assay (see above). Bodipy ® 581/591 C11 (Invitrogen), 10 µM, was used to detect lipid peroxidation. Hanks saline solution (Biochrom) without CumOOH (Sigma Aldrich) was used as a negative control, while the positive control consisted of Hanks saline solution with 50 µM CumOOH. The antioxidant control was made of growth medium with 50 µg/mL Luteolin (Cayman). Fluorescence (excitation/emission) was read at (λ) 590/632 nm (red) and at 485/520 nm (green), respectively, with a Perkin Elmer 1420 Victor plate reader using Workout 2.0 software (Dazaq Solutions Ltd.). The activity threshold was identical to the CAA assay.

### Statistics and software

Statistical analysis of the MS - data was done using EZinfo 2.0 (Umetrics, Umeå, Sweden). The markers (*m/z* and retention time pairs from LC-HR-MS data) from the extracts were compared using principal components analysis (PCA) and the data were scaled using the Pareto model. To evaluate the differences in number of active and inactive fractions between all the different assays and areas, we used Chi-square tests. The Chi-square tests (2 × 2 tables), two tailed, were done in Microsoft Excel for Mac 2011 version 14.5.7. Results from these calculations were compiled in two tables (see Supplementary Table S4 and S5). R version 3.2.2, R Core Team^52^, R studio version 0.98.1049 R Studio Team^53^ and the packages Maps and ggplot2 were used to prepare figures and plots (except PCA plot)^54,55^.

## Results

### Samples

In total we had 15 samples (i.e. 15 stations) from 3 different geographical areas: 3 samples from Finnmark, 7 from Hopen and 5 from north west of Svalbard. Sampling with the WP-2 plankton nets yielded sufficient biomass to prepare extracts that could be fractionated as well as some extra material for eventual follow up analysis. However, at the stations outside Finnmark, which was the area with the lowest plankton biomass, we had to do three - four hauls from each station to get sufficient biomass. All other stations yielded from 42–1672 grams (See Supplementary Table S6, and supplementary dataset 1). We considered 8–10 grams (wet weight) of these sample types to be the minimum amount of biomass needed for Flash - fractionation and analysis. In Finnmark, four hauls yielded 8.7 grams of wet biomass, i.e. just enough to produce the approximately 1.5 grams of dried material needed for Flash - fractionation and subsequent bioactivity testing. The stations closest to the ice edge and within the ice were rich in phytoplankton, as indicated by high *in vitro* chlorophyll *a* values (see Supplementary Fig. S3).

### Species composition

**Figure 2:**
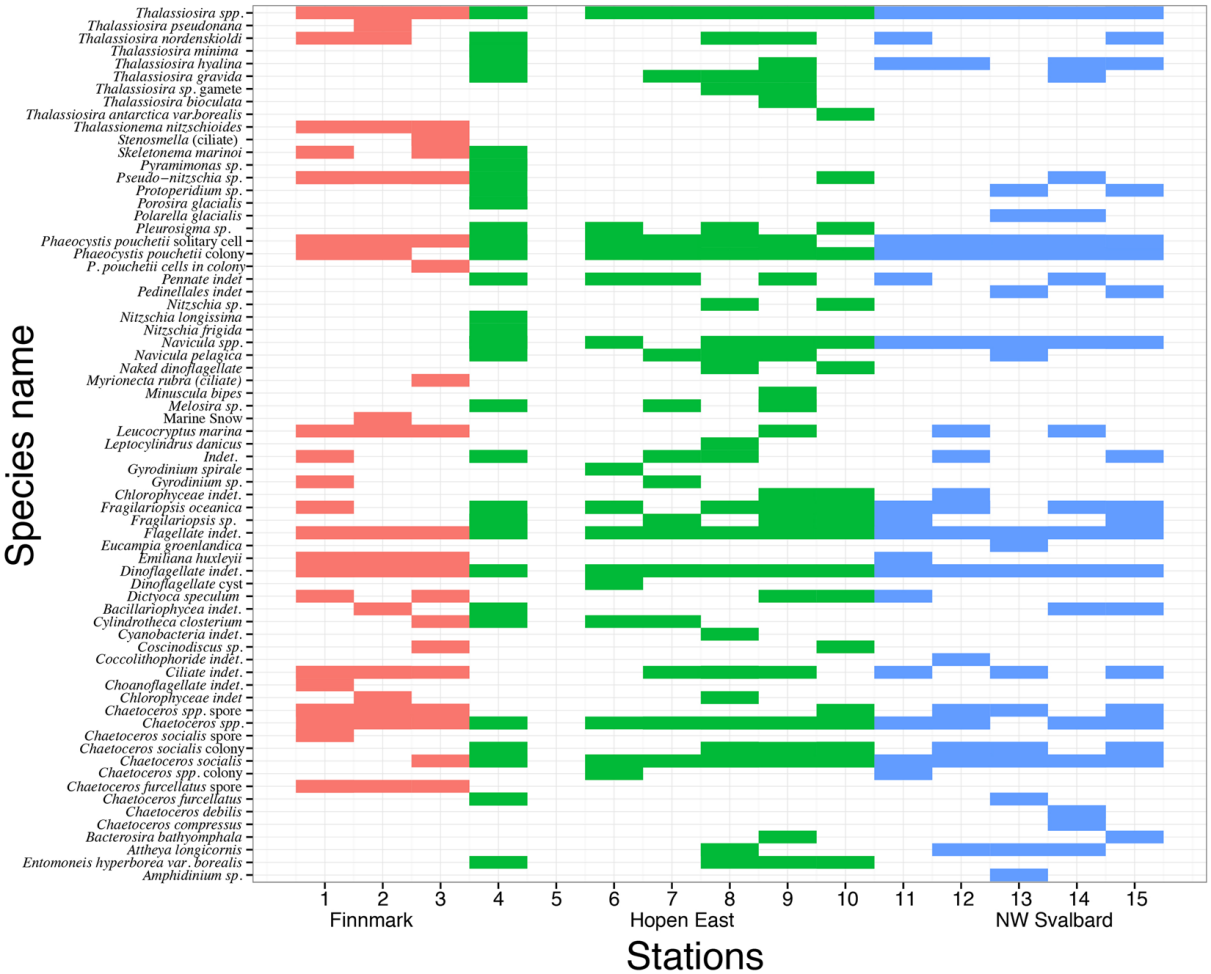
Observed presence/absence of species in water samples collected at the sampling stations from the different areas. The x - axis represents station number, while the y – axis shows species, ordinated alphabetically and by station number. The three colours represents sampling area and colour fill/no fill indicates presence/no presence (red: Finnmark, green: Hopen East and blue: NW Svalbard). Note that the position of station 4 and 5 was identical (the WP-2 sample from this station was split into a zooplankton and phytoplankton component, and the zooplankton part does not contain phytoplankton species, see methods section). Note also that “marine snow” is *not* a species, but rather aggregates of particulate matter. In addition, keep in mind that these observations were based on Niskin bottle samples, not the biomass samples.

Species observations were made from samples collected with Niskin 5-L water bottles. We observed 15 to 31 different phytoplankton species at each station (see Fig. 2), although abundance and biomass were very different between the areas (see Supplementary Fig. S3 and Supplementary Table S6). Note that the net mesh size (180 *μ*m) had an impact on phytoplankton species caught in the net. *Phaeocystis pouchetii* and various *Thalassiosira* spp. were observed at all stations. The diatoms *Chaetoceros socialis, Fragilariopsis oceanica* and *Navicula* spp. were observed at 13, 11 and 11 different stations respectively. We observed unidentified dinoflagellates at all stations, though they were not abundant. There was a clear difference in species composition between the samples collected from the coast of Finnmark (stations 1, 2 and 3) and the other samples. The biomass samples from Finnmark were composed of zooplankton, mainly the copepod *Calanus finmarchicus*, with no visible amounts of phytoplankton (see Supplementary Fig. S2). Despite low phytoplankton abundance, the quantitative samples from the same area though allowed us to determine the species composition and measure the biomass of the heavily grazed phytoplankton community. From the two other areas, Hopen East and Svalbard West, most of the samples contained exclusively phytoplankton with no visible zooplankton (stations 4, 7, 8, 9,10, 11, 12 and 15). Stations 5, 6, 13 and 14 were dominated by phytoplankton, but contained some zooplankton as well. The zooplankters were mainly copepods, with 1–2 of the arrow worm species *Parasagitta elegans* present.

### Oceanographical data

We mapped the temperature and salinity properties of the areas we sampled (see Supplementary Fig. S1 for temperature vs. salinity plots). The three areas were somewhat different regarding temperature and salinity, with the southernmost area being the warmest with temperatures up to ca. 5 °C, while the samples from the ice were as cold as −1.7 °C, i.e. close to the freezing point of seawater at this salinity. The salinity range was narrow and ranged from 34 to 35^0^/_00_. Outside Finnmark and Hopen, we found relatively homogenous water masses, while north west of Svalbard there were two separate water types, characterized by fresher, less saline surface water close to the melting ice edge and warmer water underneath.

### Bioactivity

**Figure 3:**
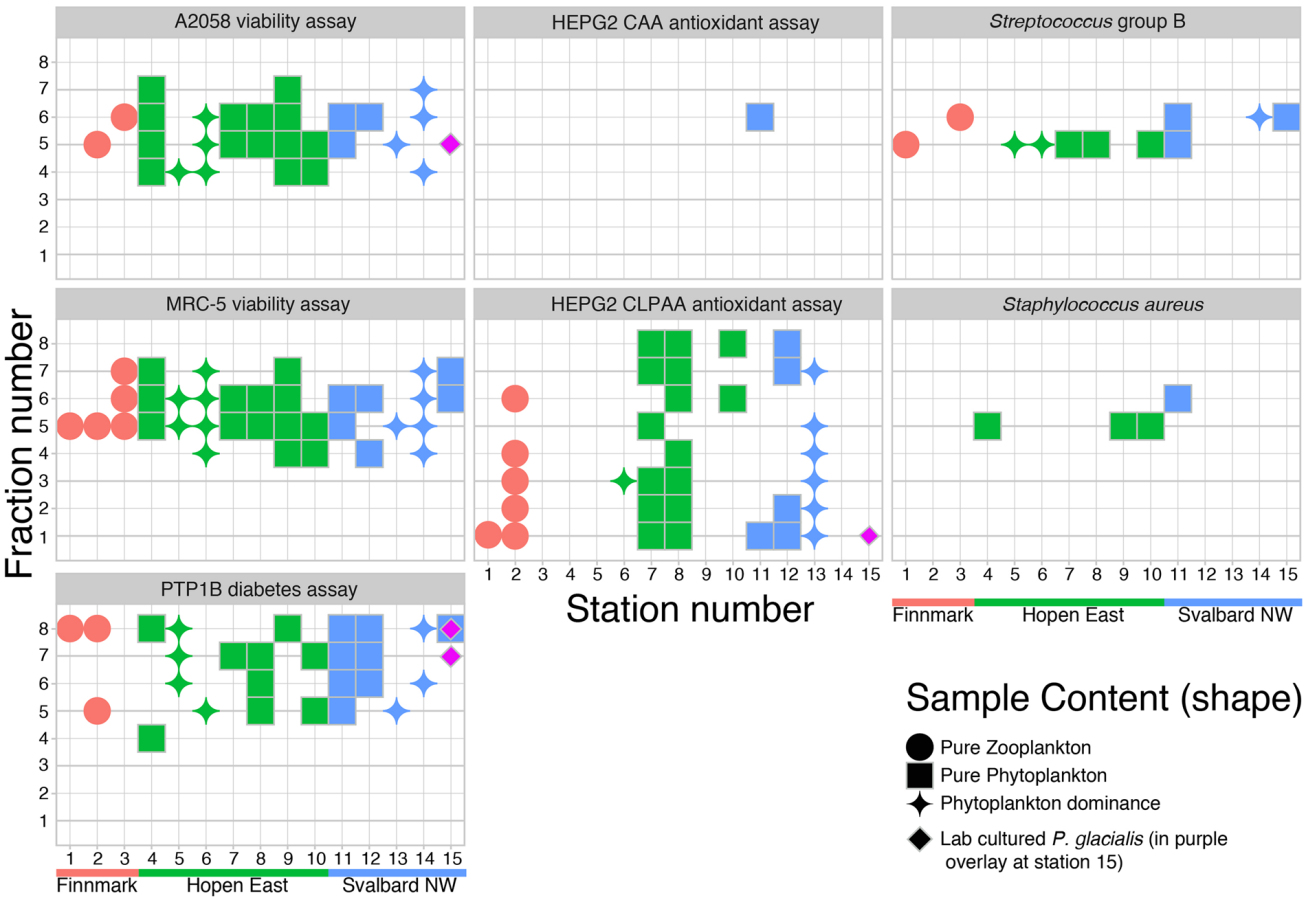
Overview of all the active fractions or “hits” in each assay in relation to the three sampling areas, sample content, station number (1–15) and fraction number (1–8) Station number is on the x - axis, while fraction number is found on the y - axis. The text in grey panels shows assay name. Red colour denotes Finnmark (stations 1–3), green colour is Hopen East (stations 4–10) and blue colour is Svalbard North West (stations 11–15). One sample per station, sample number and station numbers are the same. Geometric shapes indicate sample content: circles are solely zooplankton; squares are solely phytoplankton, while the stars denote samples dominated by phytoplankton with some zooplankton present. Please note that photobioreactor cultured *P. glacialis* activity is shown as purple overlay at station 15. Activity thresholds for the different assays are found in the methods section. The assay result category “Weak active” (see Supplementary Table S2) is not included in this figure. Note that only the PTP1B hits that were inactive in the TC-PTP1B assay are included in the figure. T-cell PTP1B inhibition (activity) is not desired and therefore the samples active in both the PTP1B assay and the TC-PTP1B assay are not interesting for anti-diabetic purposes.

Bioactivity was detected in all 3 areas, at all 15 stations/samples (see Fig. 3), but varied between the different bioassays. The assays that gave the highest number of hits were the viability assays A2058 (melanoma cancer) and MRC5 (normal lung fibroblasts), and CLPAA (Cellular lipid peroxidation), while the CAA (intracellular antioxidant activity) assay showed only one hit (see Supplementary Table S2).

No activity was found in our samples in the ABL kinase and PKA kinase (kinase inhibition) assays, against *E. coli* and *E. faecalis*, nor against biofilm forming *S. epidermidis* (see Supplementary Table S2 for details, or supplementary dataset 1). The highest overall hit rate was obtained in samples from the Hopen East area where 14.9% of sample fractions were active, while the samples from Finnmark and Svalbard had 10.1 and 10.6% active fractions, respectively. The assay result category “Weak active”, i.e. fractions just below the threshold for “active”, are reported in supplementary Table S2.

In the assay against *Staphylococcus aureus* (anti - bacterial) we found activity in samples 4, 9 and 10 from Hopen East and 11 from Svalbard North West. None of these samples contained zooplankton (see Supplementary Fig. S2). Antibacterial activity against *Streptococcus* group B was detected in samples from all areas and at a total of different 10 stations.

There was considerable activity against malignant human melanoma A2058 cells in all extracts except from the station 1 and 15 samples. Note that a fraction of the diatom photobioreactor - monoculture extract was also active against A2058 cells (this is seen as overlay at station 15). In addition, all samples had one or more fractions with activity against normal lung fibroblasts, MRC5. At some stations the anti-cancer activity was stronger and was detected across several fractions (see Supplementary Table S3). In the biochemical diabetes assay (PTP1b) we found activity in fractions from all stations, except station 3. All fractions were also screened with the TC-PTP counter screen assay. This test revealed that as many as 26 fractions were active against PTP1b, while inactive against counter screen T-cell PTP1b assay. In the cellular antioxidant assay (CAA) there was only one active fraction and it was from the extract from station 11, north west of Svalbard. In the cellular lipid peroxidation assay there was detectable bioactivity across all fractions from 1–8, and at 9 of the 15 stations (see Supplementary Table S2 and S3). These 9 stations were from all three areas and bioactivity was found both in the zooplankton samples and in the phytoplankton samples.

To check for significant differences in the number of active fractions between assay and areas (plus *P. glacialis*) and gross sample content, we applied multiple 2 × 2 Chi-square tests (two tailed, without Yates correction) with number of active and inactive in each different assay from each area/site (see Supplementary Table S4). The bioassay results from the monocultures of *P. glacialis* yielded significantly different number of hits (*p* = <0.05) in 10 of the 21 bioassays and area/site combinations (see Supplementary Table S4 for Chi-Square results and Table S3 for an overview of active vs. station, assay, area and fraction number). Between the field samples we found only one statistically significant difference at the *p* = 0.05 level: There was a significant difference in the number of active and inactive in the A2058 cancer assay between Hopen and Finnmark (*p* = 0.02). We did not find statistically significant differences in the number of active and inactive between zooplankton and phytoplankton samples (*p* = 0.19, see Supplementary Table S5), although there were a higher number of hits in the phytoplankton samples.

### Metabolomic profiling

**Figure 4:**
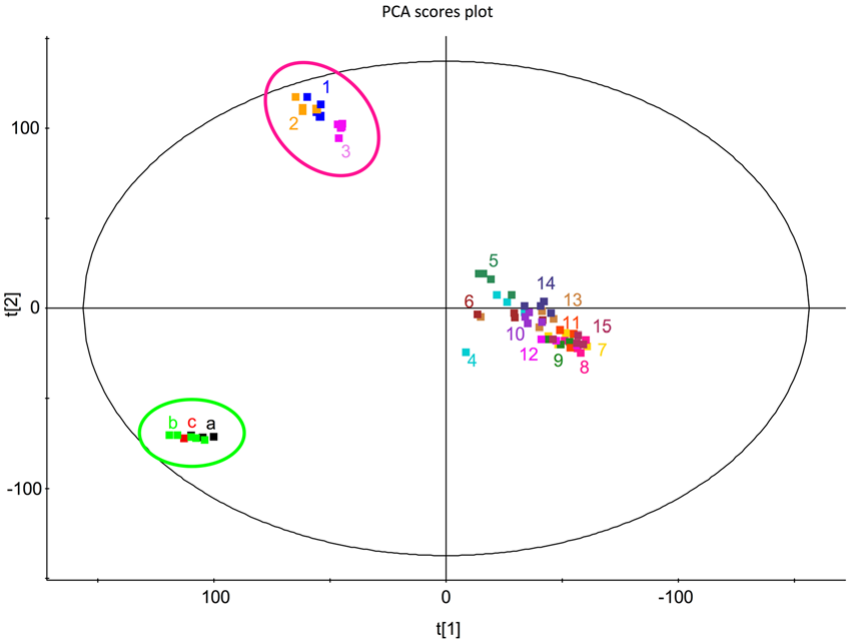
PCA - ordination of metabolites present in the crude extracts from all stations and from mass cultivated *P. glacialis*. The green circle highlights the three different reference samples (**a**,**b** and **c**) of *P. glacialis* monoculture, while the pink circle shows extracts from stations 1–3, the area near the coast of Finnmark where samples were mainly containing *Calanus* spp. zooplankton. Samples 4–15 grouped relatively close, while the two other sample groups were distinctly different.

To evaluate the chemical similarity between extracts, we compared the metabolic profiles using a Principal component analysis (PCA). The analysis revealed three distinct groups (see Fig. 4). One group contained the three samples from the coast of Finnmark, the other small group contained the three batches of *P. glacialis* monoculture (batch a, b and c), and both of these groups were distinctly different from the other samples. All samples with high contents of microalgae grouped in a larger, more loosely connected group (samples from stations 4–15).

## Discussion

To sample bulk multispecies plankton for bioactivity is uncommon, but this and similar approaches hold, in our opinion, great promise^56^. It could allow access to a vast chemical diversity, and also bypass time and labour intensive isolations and cultivations. In this pilot study, the bioassay and metabolite comparison of field samples versus a monoculture of *P. glacialis* seems promising. We identified higher bioactivity levels in all field samples than in the photobioreactor - cultivated samples of *P. glacialis*. The bioassays were applied mainly as a tool to identify whether this approach to biodiscovery could be fruitful, and the results clearly shows that it can. Furthermore, PCA ordination supported these findings. It also showed that the metabolite profiles of extracts from field samples were very different from the *P. glacialis* samples cultivated in an outdoor 6000-L photobioreactor. In addition, the site close to the coast of Finnmark, with mainly zooplankton in the samples, yielded different metabolite profiles than the other two field sites. The latter two were in fact both ice - edge algal blooms, and apparently somewhat similar despite being on opposite sides of Svalbard. These different plankton “situations” indicated different succession stages that are qualitatively different regarding species compositions and abundances. This shows that it is important to strive to find environments with more different characteristics, e.g. fjords, estuaries, open water and so on. Still, all field samples were highly bioactive.

The presence of bioactivity at all stations and in all samples was not surprising, since it is well known that planktonic organisms and especially phytoplankton can be highly bioactive or even toxic, and since some of the bioactive compounds may accumulate in the marine food chains^57–59^. However, explaining the nature and origin of the bioactivity we identified is currently impossible, and requires further work. The diversity of organisms found in our plankton net samples is probably mirroring the diversity in the given size range at the given water - column. It ranges from small phytoplankton cells, chains of cells and colonies to their grazers and the planktonic predators hunting the grazers except those able to evade the net by swimming away. The net we used for harvesting biomass had a larger mesh size than the smallest phytoplankton, although the net easily clogged, and then probably retained even some of the smallest plankton, such as bacteria and picoplankton. This should ideally be investigated in further detail, since marine bacteria are known to be an important source of bioactive compounds^60^.

Regarding areas we found only one statistically significant difference in the hit rate between the three areas. The A2058 cancer assay yielded significantly more activity in samples from the area near Hopen than outside Finnmark. Nevertheless, as seen in Fig. 3 and supplementary Tables S2 and S3 the bioactivity was not uniformly distributed between areas and stations.

Regarding the choice of assays, there are several considerations. The most obvious ones are time, cost and logistical constraints. Furthermore, the assays we have chosen are aimed at biodiscovery in a human medicine perspective. If we e.g. were to search for aquaculture relevant medicines, clearly a different set of assays would be more relevant. The assays we applied can be categorized as two types of assays: phenotypic cellular assays and biochemical assays. In the cancer screening we used both types (see methods section), for diabetes a biochemical assay, while in the antioxidant screening we only used phenotypic assays, although two very different types that detects different aspects of antioxidant activity (CAA and CLPAA). The most important pro with phenotypic assays with living cells like CAA and CLPAA is that they naturally account for uptake of active compounds^50,61^.

In the cancer assays we observed a high number of active fractions. However, this was no surprise. In 1999, Miralto, *et al*.^62^ showed that a diatom diet could have an insidious effect on copepod reproduction, and on Caco2 cells. Later, it has been shown that microalgae and diatoms are capable of producing oxylipins as a response to grazing^63–65^. The copepod grazers on the other hand seem to respond to the oxylipin presence by activating or up - regulating defence mechanisms like detoxification enzymes production^66^. Although some authors dispute that these compounds have evolved as a defence mechanism^67^, the compounds certainly exists. In diatoms, this group of oxygenated fatty acid derivatives is both diverse and complex^68^. Our sample handling, causing mechanical stress by e.g. squeezing cells onto a mesh while harvesting them, can probably trigger this response. The resulting oxylipin production will, if the oxylipins do not decompose during the extraction process, probably cause a broad and unspecific response in e.g. the A2058 and MRC5 assay (and also most other cells). In drug discovery, some of this activity, caused by e.g. volatile oxylipins, is not necessarily desired and will ideally be quickly de - replicated downstream if present. With modern HR-MS coupled HPLC techniques, this is indeed possible^69^. On the other hand, it is likely that the extraction protocol applied, with freeze - drying, a long extraction and evaporating at 40 °C for hours leave only the less volatile compounds like non-volatile oxylipins behind. Bioactivity profiles of most non-volatile oxylipins are not described^68^. Therefore, the observed activity is still of interest.

It would be very interesting to detect compounds that have specific activity. However, of the assays we applied it is only the kinase assays and the PTP1B that are target-based, i.e. that a compound has to interact with a pre-defined molecular target in order to be recorded as active in the assay. That said, biochemical assays need to be followed up with cell-based assays to get information about bioavailability, ability to cross membranes etc. A well - known example of a toxin with specific activity is the dinoflagellate toxin maitotoxin, which act directly on Ca^2+^ channels in animals^70^. Dinoflagellate toxins are often extremely potent, and “brown tides” or blooms caused by toxic dinoflagellates can be dangerous both to fish, marine mammals and people^71^. We did observe dinoflagellates at all stations, but most of these were difficult to identify and abundance was not high, but considering the potency of dinoflagellate toxins, they cannot be ruled out as source. Usually, such activity causes concerns since they may be dangerous to people, fish and other animals^72^. But potent toxins can potentially be developed into medicines, and several studies have been carried out to investigate this potential^73^.

We observed antioxidant activity in the cellular lipid peroxidation assay (CLPAA), but only one hit in the cellular antioxidant assay (CAA). Both assays are cell - based, but measures different aspects of antioxidant activity. The CAA assay measures the inhibition of intracellular reactive oxygen species (ROS) activity while the CLPAA assay measures inhibition of intracellular lipid peroxidation^74^. Diatoms are naturally rich in antioxidants and there are many known compounds with antioxidant properties in diatoms, especially among the many pigments^75–77^. The cellular CAA and CLPAA assays do not necessarily pick up all of these, because antioxidant activity is heterogeneous and there are many different ways of measuring such activity^61^. Many phytoplankton species contain a group of compounds called “mycosporine like amino-acids” (MAA’s) that have photo - protective properties^78,79^. There was an abundance of *P. pouchetii* and various diatoms in our samples, and both *P. pouchetii* and diatoms like e.g. *Porosira glacialis* have been shown to contain MAA’s^80–82^. The composition of pigments and MAA’s is influenced by light conditions, and exposure to high light intensities and UV- light may trigger production of certain pigments or alter the ratios between pigments^80^ or amount of MAA’s^81^, although this varies between species.

Antibacterial activity in phytoplankton has been known for many decades^83^, and diatoms like e.g. *Skeletonema costatum* sensu lato show a quite broad range of antibacterial activity as tested and reviewed by Naviner, *et al*.^84^. We observed activity against *Streptococcus* group B and *S. aureus*. Since the sea is teeming with bacteria and other microbes^85^, one could expect it to be common for planktonic organisms to produce antibiotics to protect them from being attacked by bacteria and fungi.

The spatial distance between the sampling areas in this study was considerable with approximately 1,000 km between Finnmark and Svalbard North West and 600 km between Hopen and Finnmark (see Fig. 1). We did identify differences in water temperature and salinity (see Supplementary Fig. S1). This difference in physical conditions may be interesting since some of the species we detected in this study, e.g. *Chaetoceros socialis*, *Porosira glacialis* and *Skeletonema marinoi* have been reported to respond chemically to changes in e.g. temperature and light^3^. Other authors have also reported changes in bioactivity due to variation in environmental conditions like temperature^86^ and light^87^. Biologically, the content of our samples and the chlorophyll measurements indicated differences in spring - bloom stage, with the Finnmark coast probably being in a post bloom stage, the Hopen area in a late bloom stage while we faced a vigorous ice-edge bloom in the Svalbard North West area (See Supplementary Figure S3). This has implications for the nutrient availability for the phytoplankton, and this may in turn affect phytoplankton bioactivity^88^. Furthermore, low nutrient availability and low temperature could cause stress and hence change the bioactivity profiles. Thus, the areas were qualitatively different from each other, regarding both physical and biological factors. The bioactivity analyses, although not statistically significant at the *p* = 0.05 level, also indicated non-identical situations. The variation was in our data seemingly spatial - variations, especially in abundance, possibly due to difference in the succession stages of the spring bloom. Nevertheless, the PCA analysis indicated that there were small differences in metabolomic profiles between the samples from Hopen and north west of Svalbard.

The goal of this study was to demonstrate the use of bulk sampling of plankton for biodiscovery, and not identification of specific compounds *per se*. Still, it was important to add a proof of concept including a convincing dereplication strategy. The presence of promiscuous bioactive compounds in extracts of living matter is well known, and the presence of such compounds in screening campaigns are often referred to as PAINS (pan-assay interference compounds)^89^. We therefore investigated the HR-MS data of selected samples for well known, abundant and commonly observed compounds. We found phosphatidylcholines to be present in many of the bioactive extracts. One example is *m/z* 496.3396 ([M + H]^+^), corresponding to a phosphatidylcholine  with the elemental composition C_24_H_50_NO_7_P. However, when comparing the bioactivity profile and the presence of phosphatidylcholines, it is clear that the observed bioactivity can not be explained by the phosphatidylcholines alone, so it is clear that there are more bioactive compounds than just the abundant phosphatidylcholines.

We suggest that the relatively high diversity with respect to environmental conditions and species composition could increase the possibility of finding novel bioactivity. Sampling at other conditions, sites and areas could easily increase the chemical diversity in samples. We also hypothesize that metabolic pathways that would otherwise be silent under controlled growth in monocultures, are activated in the field samples.

### Conclusions

We detected bioactivity in all bulk samples from the 15 stations at the three sites we investigated. This is interesting for biodiscovery purposes. The sampling was done over a rather large area with only a few days separating the first samples from Finnmark and last samples from west Svalbard. This resulted in qualitative variation in species composition, biomass concentration and physical conditions (temperature, salinity profile and light regime), and these differences were probably contributing to some of the variability in the bioactivity we observed. This study indicates that it is possible to access extensive chemical diversity by sampling complex marine plankton communities at different locations and time-points.

## Electronic supplementary material

Supplementary material

Supplementary Dataset 1

Supplementary Dataset 2
