# Supplementary material for: Field sampling marine plankton for biodiscovery
Source: Sci Rep. 2017 Nov 20;7:15863. doi: 10.1038/s41598-017-15980-8 (PMC5696511; doi:10.1038/s41598-017-15980-8)
Supplement: Supplementary file 1 — Supplementary material [file 41598_2017_15980_MOESM1_ESM.pdf]

Supplementary material

Field sampling marine plankton for biodiscovery

Richard Andre Ingebrigtsen [1]*, Espen Hansen [2], Jeanette Hammer Andersen [2], Hans Christian Eilertsen[1]

[1] Norwegian College of Fishery Science, UiT - The Arctic University of Norway

[2] Marbio, UiT - The Arctic University of Norway

*** Corresponding author**

Richard Andre Ingebrigtsen
Norwegian College of Fishery Science, UiT - The Arctic University of Norway, 9019 Tromsø, Norway

Supplementary figures S1 - S3

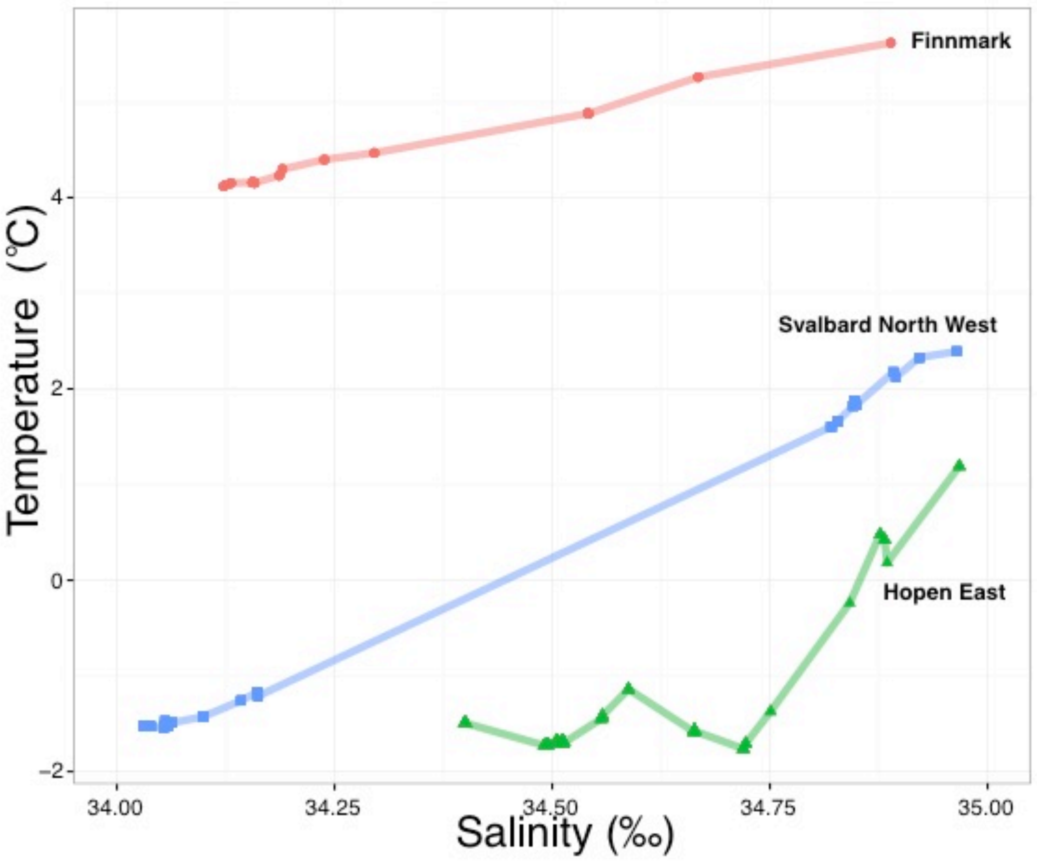

Supplementary Figure S1. Temperature and salinity plot for the three study sites. Colours indicate sampling area: red is Finnmark, blue is Svalbard North East and green is Hopen East. The TS data from all 15 stations were measured at depths: 0, 5, 10, 20, 50 and 100 meter (Please note that at Svalbard North West, sampling was only down to 50 meters).

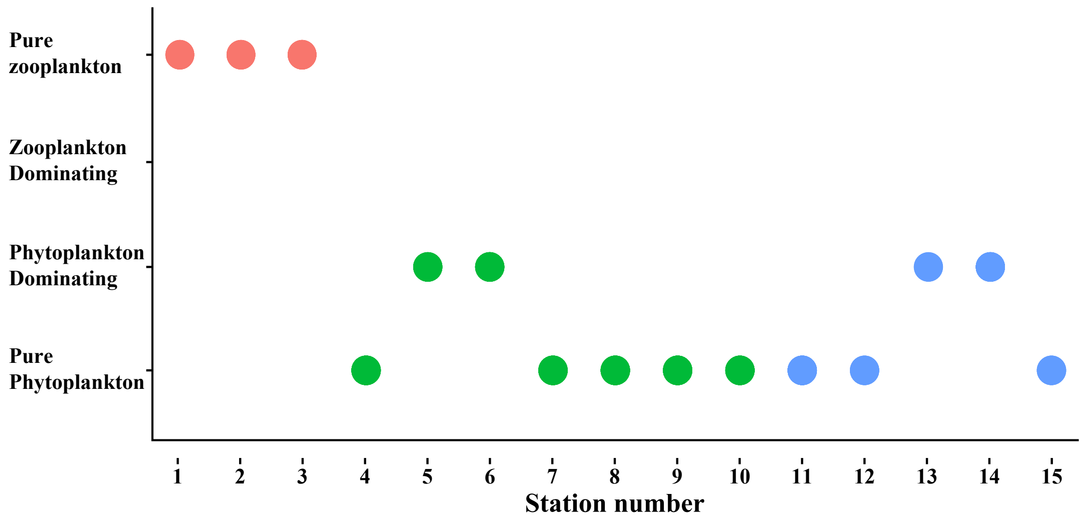

Supplementary Figure S2. Zooplankton vs. phytoplankton content of net - samples. The sample content was qualitatively assessed after sampling and freeze - drying. Note that the mesh size of the WP-net used for net samples was 180 μm while the phytoplankton single cell size ranged from 5 - 200 μm, although some were chain or colony forming species. In the "pure phytoplankton" category there was no visible large zooplankton, while in the "pure zooplankton" there was no visible coloration due to phytoplankton. No samples were dominated by zooplankton, while there were 4 samples with the majority of biomass consisting of phytoplankton, but with some zooplankton presence. The zooplankton observed was predominantly the very common species *Calanus finmarchicus*, while the phytoplankton community was diverse (see Fig. 3).

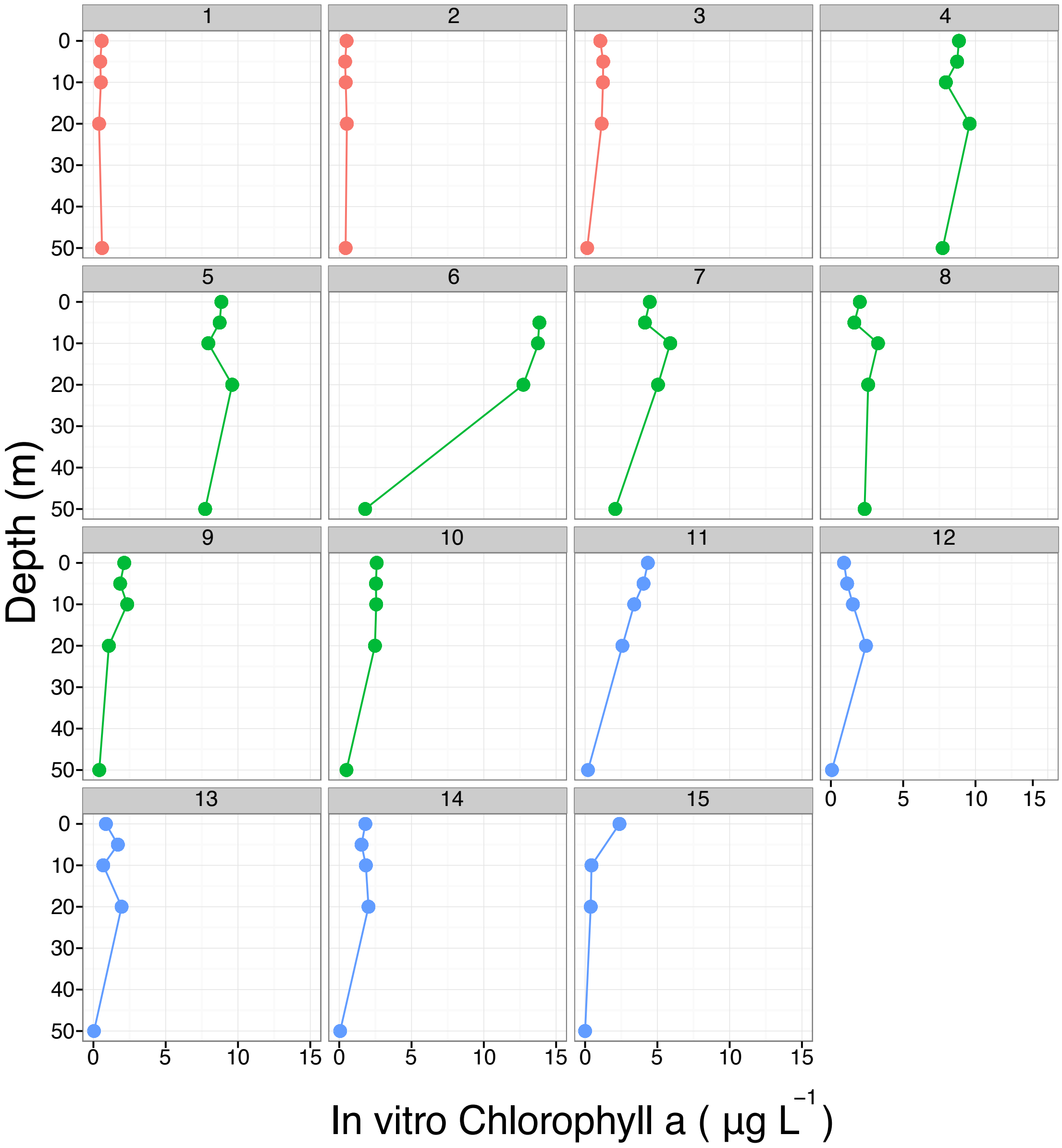

Supplementary Figure S3. *In vitro* chlorophyll *a* measurements at 0, 5, 10, 20 and 50 meters

The figure shows *in vitro* chlorophyll *a* (in this case a proxy for phytoplankton biomass) from samples taken at five fixed depths (0, 5, 10, 20 and 50 meters) at each station. Note that station 4 and 5 is the same since the net sample biomass from this station was separated into one zooplankton component and one phytoplankton component. The three colours indicate sampling area; red (station 1-3) is the coast of Finnmark, green (station (4-10) is East of Hopen while blue (station 11-15) is north west of Svalbard (see Fig. 1).

Supplementary Tables S1 - S6

Supplementary Table S1. Solvent gradient applied for FLASH column fractionation of the extracts into 8 fractions depending on the polarity. The solvent mix numbers denote percentage (%) of each solvent for each step (fraction). Note that fraction 5 and 6 is the same mix. Fractions 1 – 6 consisted of water – methanol gradient, while fractions 7 – 8 consisted of a methanol – acetone gradient.

**Fraction**	1	2	3	4	5	6	7	8
**Water**	95	75	50	25	0	0	0	0
**MeOH**	5	25	50	75	100	100	50	0
**Acetone**	0	0	0	0	0	0	50	100

Supplementary Table S2. Overview of all assay results (n =2008) The numbers (1-8) denote how many fractions were active, inactive or weak active in each assay. There were 8 fractions per station/sample. Assay name (left column), sampling area (top), station number (middle heading) and Zooplankton/Phytoplankton content (lower heading). Zooplankton presence was graded qualitatively/visually where “Z” denotes only zooplankton present, “M” Phytoplankton dominance (zooplankton present) and “P” was solely phytoplankton. There were no samples dominated by zooplankton with visible amounts of phytoplankton present at the same time. Assay outcome is shown in the results part as number of fractions being active, inactive or weakly active (see methods section for activity thresholds). Note that the TC – PTP1B assay is a counter screen of the PTP1B assay. T-cell PTP1B inhibition (activity) is not desired and therefore the samples active in both the PTP1B assay and the TC-PTP1B assay are not interesting for anti-diabetic purposes.

Area	Finnmark			Hopen East							Svalbard North West					*Porosira glacialis*		
**Assays**	1	2	3	4	5	6	7	8	9	10	11	12	13	14	15	Pg_1	Pg_2	Pg_3
	Z	Z	Z	P	M	M	P	P	P	P	P	P	M	M	P	P	P	P
A2058_MTT																		
Active		1	1	4	1	3	2	2	4	2	2	1	1	3		1	1	
Inactive	8	7	7	4	7	5	5	6	4	6	6	7	7	5	8	7	7	8
Weak active							1											
ABL_KINASE																		
Inactive	8	8	8	8	8	8	8	8	8	8	8	8	8	8	8	8		8
ECOLI																		
Inactive	8	8	8	8	8	8	8	8	8	8	8	8	8	8	8	8	8	8
EFAECALIS																		
Inactive	8	8	8	8	8	8	8	8	8	8	8	8	8	8	8	8	8	8
HEPG2_CAA																		
Active											1							
Inactive	8	8	8	8	8	8	8	7	8	8	7	8	8	8	8	8	8	8
Weak active								1										
HEPG2_CLPAA																		
Active	1	5				1	6	7		2	1	4	6				1	
Inactive	5	3	8	8	8	4	2	1	7	3	6	2	1	7	8	8	5	8

Weak active	2					3			1	3	1	2	1	1			2	
MRC-5_MTT																		
Active	1	1	3	3	2	4	2	2	4	2	2	2	1	4	2	1	1	1
Inactive	5	5	5	4	5	4	6	6	4	6	6	6	7	4	6	7	7	7
Weak active	2	2		1	1													
PAERUGINOSA																		
Inactive	8	8	8	8	8	8	8	8	8	8	8	8	8	8	8	8	8	8
PKA_KINASE																		
Inactive	8	8	8	8	8	8	8	8	8	8	8	8	8	8	8	8	8	8
PTP1B																		
Active	4	2	3	5	5	3	3	3	4	3	4	3	1	3	3	2	4	2
Inactive	4	5	5	3	3	5	5	5	4	5	4	5	6	4	5	4	4	5
Weak active		1											1	1		2		1
SAUREUS																		
Active				1					1	1	1							
Inactive	8	8	8	7	8	7	7	8	7	7	7	8	8	8	8	8	8	8
Weak active						1	1											
BIOFILM																		
Inactive	8	8	8	8	8	8	8	8	8	8	8	8	8	8	8	8	8	8
STREPTB																		
Active	1		1		1	1	1	1		1	2			1	1			
Inactive	7	8	6	7	7	7	7	7	8	7	6	8	8	7	7	8	8	8
Weak active			1	1														
(TC-PTP1B)																		
Active	3		3	3	2	2	2		3	1				2	2	3	1	2
Inactive	5	8	5	5	6	6	6	8	5	7	8	8	8	6	6	5	6	5

Weak active																	1	1
**Total**	**112**	**112**	**112**	**112**	**112**	**112**	**112**	**112**	**112**	**112**	**112**	**112**	**112**	**112**	**112**	**112**	**104**	**112**

Supplementary Table S3. All active ("hits") in relation to sampling area, station and fraction number. "1" means it is a hit at that particular station, fraction and assay, while "-" means no hit. "Weak active" is not listed this table (n=203). "NWS" denote north west of Svalbard, Stations are 1-15, while monoculture of *Porosira glacialis* is given the "station numbers" Pg1-Pg3.

**Assay name**	**Station / Fraction**	**Finnmark**			**Hopen East**							**NWS**					***Porosira glacialis***			**Tot.**
		**1**	**2**	**3**	**4**	**5**	**6**	**7**	**8**	**9**	**10**	**11**	**12**	**13**	**14**	**15**	**Pg1**	**Pg2**	**Pg3**	**SUM**
**CLPAA**	**1**	1	1		-	-	-	1	1	-	-	1	1	1	-	-	-	1	-	8
**CLPAA**	**2**	-	1	-	-	-	-	1	1	-	-	-	1	1	-	-	-	-	-	5
**CLPAA**	**3**	-	1	-	-	-	1	1	1	-	-	-	-	1	-	-	-	-	-	5
**A2058**	**4**	-	-	-	1	1	1	-	-	1	1	-	-	-	1	-	-	-	-	6
**CLPAA**		-	1	-	-	-	-	-	1	-	-	-	-	1	-	-	-	-	-	3
**MRC-5**		-	-	-	-	-	1	-	-	1	1	-	1	-	1	-	-	-	-	5
**PTP1B**		-	-	-	1	1	-	-	-	-	-	-	-	-	-	-	1	1	1	5
**TC-PTP**		-	-	-	-	1	-	-	-	-	-	-	-	-	-	-	1	1	1	4
**A2058**	**5**	-	1	-	1	-	1	1	1	1	1	1	-	1	-	-	1	1	-	11
**CLPAA**		-	-	-	-	-	-	1	-	-	-	-	-	1	-	-	-	-	-	2
**MRC-5**		1	1	1	1	1	1	1	1	1	1	1	-	1	1	-	1	1	1	16
**PTP1B**		1	1	1	1	1	1	1	1	1	1	1	-	1	-	-	-	1	-	13
S. aureus		-	-	-	1	-	-	-	-	1	1	-	-	-	-	-	-	-	-	3
***Strept.* Gr.B**		1	-	-	-	1	1	1	1	-	1	1	-	-	-	-	-	-	-	7
**TC-PTP**		1	-	1	1	1	-	1	-	1	-	-	-	-	1	-	1	-	1	9
**A2058**	**6**	-	-	1	1	-	1	1	1	1	-	1	1	-	1	-	-	-	-	9
**CAA**		-	-	-	-	-	-	-	-	-	-	1	-	-	-	-	-	-	-	1
**CLPAA**		-	1	-	-	-	-	-	1	-	1	-	-	-	-	-	-	-	-	3
**MRC-5**		-	-	1	1	1	1	1	1	1	-	1	1	-	1	1	-	-	-	11

**PTP1B**		1	-	1	1	1	1	1	1	1	1	1	1	-	1	1	-	-	-	13
S. aureus		-	-	-	-	-	-	-	-	-	-	1	-	-	-	-	-	-	-	1
***Strept*. Gr.B**		-	-	1	-	-	-	-	-	-	-	1	-	-	1	1	-	-	-	4
**TC-PTP**		1	-	1	1	-	1	1	-	1	1	-	-	-	-	1	-	-	-	8
**A2058**		-	-	-	1	-	-	-	-	1	-	-	-	-	1	-	-	-	-	3
**CLPAA**		-	-	-	-	-	-	1	1	-	-	-	1	1	-	-	-	-	-	4
**MRC-5**	**7**	-	-	1	1	-	1	-	-	1	-	-	-	-	1	1	-	-	-	6
**PTP1B**		1	-	1	1	1	1	1	1	1	1	1	1	-	1	1	1	1	-	15
**TC-PTP**		1	-	1	1	-	1	-	-	1	-	-	-	-	1	1	1	-	-	8
**CLPAA**	**8**	-	-	-	-	-	-	1	1	-	1	-	1	-	-	-	-	-	-	4
**PTP1B**		1	1	-	1	1	-	-	-	1	-	1	1	-	1	1	-	1	1	11
**Total**	**8**	10	9	11	16	11	14	16	15	16	12	13	10	9	13	8	7	8	5	203

Supplementary Table S4. Chi-Square tests results (2x2 tables) of the number of active versus inactive in each assay and area and the *Porosira glacialis* monoculture (n =2008). Significant results are marked with an asterisk (*). Note that all except one of the significant results are found with the *P. glacialis*.

Assay	Area	Finnmark	Hopen	Svalbard NW	*P. glacialis*
A2058	**Finnmark**	-	0.02*	0.31	1.00
	**Hopen**	0.02*	-	0.10	0.02*
	**Svalbard NW**	0.31	0.10	-	0.31
	P. glacialis	1.00	0.02*	0.31	-
MRC5	**Finnmark**	-	0.41	0.84	0.28
	**Hopen**	0.41	-	0.43	0.04*
	**Svalbard NW**	0.84	0.43	-	0.16
	P. glacialis	0.28	0.04*	0.16	-
CLPAA	**Finnmark**	-	0.65	0.74	0.04
	**Hopen**	0.65	-	0.91	0.01*
	**Svalbard NW**	0.74	0.91	-	0.02
	P. glacialis	0.04	0.01*	0.02	-
PTP1b	**Finnmark**	-	0.55	0.86	0.94
	**Hopen**	0.55	-	0.36	0.51
	**Svalbard NW**	0.86	0.36	-	0.92
	P. glacialis	0.94	0.51	0.92	-
TC-PTP1B	**Finnmark**	-	0.70	0.44	0.04
	**Hopen**	0.70	-	0.19	0.01*
	**Svalbard NW**	0.44	0.19	-	0.02*
	P. glacialis	0.04	0.01*	0.02*	-
S. aureus	**Finnmark**	-	0.70	0.44	1.00
	**Hopen**	0.70	-	0.19	0.01*
	**Svalbard NW**	0.44	0.19	-	0.02*
	P. glacialis	1.00	0.01*	0.02*	-
Strept. Gr. B	**Finnmark**	-	0.70	0.44	0.04*
	**Hopen**	0.70	-	0.19	0.01*
	**Svalbard NW**	0.44	0.19	-	0.02*
	P. glacialis	0.04*	0.01*	0.02*	-

Supplementary Table S5. Chi square test table (2x2 test) of the difference in the number of active and inactive fractions between samples containing only zooplankton, only phytoplankton or a mix where phytoplankton dominates (n =1680). Note that the results from monoculture samples (of *P. glacialis*) are not included in this test.

**Sample Content**	Phytoplankton	Phytopl. Dominant	Zoopl. Dominant	Zooplankton
Phytoplankton	-	0.701	-	0.192
Phytopl. Dominant	0.701	-	-	0.436
Zoopl. Dominant	-	-	-	-
Zooplankton	0.192	0.436	-	-

Supplementary Table S6. Overview of yields from WP2 net samples, and the corresponding dry weights.

**Station**	**Weight (grams)**	**Dry weight**	**% dry weight**
1	41	2.8	6.9
2	47	5	10.7
3	9	1.5	16.8
4	115	6.4	5.6
5	84	3.6	4.2
6	609	25.9	4.2
7	1370	46.9	3.4
8	1328	49.9	3.8
9	170	6.6	3.9
10	280	11.9	4.2
11	448	16.9	3.8
12	460	16.4	3.6
13	1672	64	3.8
14	275	10.9	4
15	458	16.3	3.6
